# Copy number evolution of conserved noncoding RNA gene families across mammals

**DOI:** 10.1007/s00335-026-10243-2

**Published:** 2026-05-22

**Authors:** Zewei Yang, Zixia Huang

**Affiliations:** 1 School of Biology and Environmental Science, University College Dublin, Dublin 4, Ireland; 2 School of Medicine, University College Dublin, Dublin 4, Ireland

## Abstract

Noncoding RNAs (ncRNAs) play essential roles in mammalian gene regulation, yet their copy number evolution across mammals remains poorly characterised. Here, using high-confidence Covariate Models (CMs) from Rfam, we applied a unified INFERNAL-based framework to systematically predict copy numbers of 588 well-curated, conserved ncRNA gene families across 60 mammalian genomes. Phylogenetic correlation tests indicated that variation in ncRNA family number and total copy numbers is largely independent of genome size, scaffold number, or assembly completeness, indicating that lineage-specific evolutionary processes, rather than genome characteristics, primarily shape ncRNA copy number diversity. Using CAFE analysis, we inferred a burst of ncRNA expansions in the eutherian ancestor, predominantly involving snRNA and H/ACA snoRNA families. In contrast, estimates of phylogenetic signals (Pagel’s λ) and evolutionary rates (σ^2^) revealed that many miRNA families exhibit lineage-specific copy number expansions. Analysis of highly variable ncRNA families further showed frequent overlap with transposable elements and distinct duplication strategies, including tandem clustering (e.g., *mir-154* and *SNORA116*) and dispersed distributions with regional tandem duplications (e.g., *5S_rRNA*). Collectively, these results provide a comparative overview of copy number variation among conserved ncRNA families in mammals. While individual predicted copies may include pseudogenised or TE-derived loci, our analyses reveal robust evolutionary patterns at the family level and provide a foundation for future studies of ncRNA duplication and diversification.

**Supplementary Information:**

The online version contains supplementary material available at 10.1007/s00335-026-10243-2.

## Introduction

For decades, much of the genome was dismissed as ‘Junk DNA’, with biological significance attributed almost exclusively to protein-coding genes (Doolittle 2013). Yet these protein-coding genes account for less than 2% of the human genome (Lander et al. 2001; Piovesan et al. 2016), leaving the vast majority of genomic regions unexplained. The persistence of noncoding DNA over millions of years of evolution suggests that at least a subset of these regions are subject to selective constraint beyond protein-coding genes. Indeed, a recent comparative genomics analysis of 240 placental mammals found that ~ 10.7% of the human genome is under evolutionary constraint, with nearly half of these bases unannotated and largely located in non-coding regions (Christmas et al. 2023). This evolving view has brought increasing attention to noncoding elements, including noncoding RNAs (ncRNAs), as components of genome biology, although their broader functional and evolutionary roles remain poorly understood.

ncRNAs are RNA molecules transcribed from the genome that do not encode proteins but can function as structural, catalytic, or regulatory elements (Eddy 2001). They are broadly classified into housekeeping and regulatory types. Housekeeping ncRNAs, including ribosomal RNAs (rRNAs), transfer RNAs (tRNAs), small nucleolar RNAs (snoRNAs), and small nuclear RNAs (snRNAs), play well-established roles in ribosome biogenesis, RNA splicing, and protein translation. Regulatory ncRNAs, such as microRNAs (miRNAs), small interfering RNAs (siRNAs), PIWI-interacting RNAs (piRNAs), long noncoding RNAs (lncRNAs), and circular RNAs (circRNAs), have been implicated in gene expression and cellular regulation in a number of well-studied systems. For example, the lncRNA *XIST* (X-inactive specific transcript) controls X-chromosome inactivation (Herzing et al. 1997), and specific miRNAs such as miR-146a and miR-155 regulate immune responses (Testa et al. 2017). In addition, *SNORD116* is an snoRNA involved in RNA processing and likely in the regulation of neural and metabolic pathways, whose loss causes Prader-Willi syndrome (Bieth et al. 2015). While these examples demonstrate that some ncRNAs can exhibit important biological roles, they are largely derived from a limited number of intensively studied genes in model organisms. The functional relevance of most annotated ncRNAs remains uncertain, particularly in a comparative evolutionary context.

Gene duplication is a fundamental evolutionary process that increases genomic content and can generate genetic variation (Innan And Kondrashov 2010). While classical examples such as the opsin and globin gene families demonstrate how duplications can contribute to functional diversification in protein-coding genes (Dulai et al. 1999; Storz 2016), the consequences of duplication for ncRNAs are less systematically understood. For ncRNAs, duplication can derive through distinct mechanisms such as whole genome duplication, segmental duplication, retrotransposition, potentially affecting copy number and genomic distribution. In some cases, duplicated ncRNAs have been associated with dosage effects or regulatory diversification, such as the expansion of tRNA genes (Hughes et al. 2023) and the vertebrate-specific mir-17 ~ 92 cluster (Mogilyansky And Rigoutsos 2013). Similarly, members of the let-7 miRNA family illustrate how paralogs can exhibit divergent expression patterns (Abbott et al. 2005; Hertel et al. 2012). However, the copy number dynamics of ncRNA repertoires, regardless of their functional status, remains poorly understood across mammals.

Bridging this knowledge gap, however, is hampered by significant methodological challenges in comparative ncRNA annotation. The prevailing alternative, using a specialised tool for each ncRNA class (e.g., MirMachine for miRNA (Umu et al. 2023), CPC2 for LncRNA (Kang et al. 2017), tRNAscan-SE for tRNA (Chan et al. 2021), presents limitations for a broad comparative study. While these tools are highly accurate within their domain, they yield data that is intrinsically incompatible. Each tool employs different algorithms, confidence thresholds, underlying principles, and often requires specific types of supporting evidence (e.g., transcriptomic data). This creates a fragmented and inconsistent annotation landscape across ncRNA classes and across species, making unified evolutionary analysis practically impossible. Furthermore, the accuracy of many specialised tools diminishes when applied beyond the model organisms they were trained on (e.g., siSPOTR for human and mouse snRNA (Boudreau et al. 2013), LncDC for mouse LncRNA (Li And Liang 2022), rendering them suboptimal for a survey across the mammalian phylogeny (Zhang et al. 2017). In consequence, the evolutionary dynamics of ncRNA copy number variation within mammals remain obscured by these inconsistent and incomplete annotations.

To overcome these limitations and generate a consistent, genome-wide dataset, we employed a unified comparative genomics approach using the INFERNAL pipeline. INFERNAL uses statistical Covariance Models (CMs) that simultaneously encode sequence conservation and secondary structure, providing a single, powerful metric for detecting diverse ncRNA families. We directly address the valid concern of poorly curated Rfam models by implementing a stringent pre-filtering strategy and parameter setting (See Materials and Methods). We exclusively used high-confidence CMs built from alignments of more than 10 sequences, ensuring robust and evolutionarily informed models. This rigorous selection mitigates noise and focuses our analysis on well-conserved, reliably annotated ncRNA families. By applying this consistent, structure-aware, and quality-controlled framework across 60 mammalian genomes, we provide a unified perspective on copy number evolution of conserved ncRNA families, establishing a foundational dataset for future functional and lineage-specific investigations.

## Materials and methods

### Mammalian genome sampling

In this study, we predicted and analysed ncRNA gene copy number in 60 mammalian genomes spanning 17 orders, representing broad ecological and phylogenetic diversity across both eutherian and non-eutherian lineages (Table S1). The dataset includes Carnivora (*n* = 10), Rodentia (*n* = 9), Primates (*n* = 9), Cetartiodactyla (*n* = 6), Chiroptera (*n* = 6), Lagomorpha (*n* = 3), Perissodactyla (*n* = 3), Eulipotyphla (*n* = 3), Monotremata (*n* = 2), Dasyuromorphia (*n* = 2), and one species each from Didelphimorphia, Microbietheria, Diprotodontia, Dermoptera, Proboscidea, Pilosa, and Cingulata. All genomes were obtained from the NCBI RefSeq repository, and were assembled at the chromosome level. Genome completeness was assessed using the BUSCO (Benchmarking Universal Single-Copy Orthologs) framework with the mammalia_db10 dataset (Simao et al. 2015). Complete BUSCO scores ranged from 92.8% to 99.7% (mean = 98.3%), indicating the high quality and assembly completeness of these genomes. Details on species representation, genome assembly versions and statistics, and assembly completeness are available in Table S1.

### Curated Rfam covariate model (CM) selection

To ensure the accuracy and reliability of ncRNA predictions, we created a curated set of high-confidence CMs from the Rfam database (v15) (Ontiveros-Palacios et al. 2025). Rfam CMs are derived from multiple sequence alignments that capture both sequence conservation and secondary structure, but models built from shallow alignments may be poorly curated or represent lineage-restricted RNAs. Currently, the Rfam database contains 4,177 CMs, including 2 tRNA, 14 rRNA, 809 snRNA/snoRNA, 1,598 miRNA, 218 lncRNA, 524 Cis-reg, and 1,012 miscellaneous RNA classes. To improve robustness, we excluded 2,742 (65.6%) out of 4,177 CMs that were built from alignments with fewer than 10 sequences, resulting in a final set of 1,435 high-confidence CMs (34.4%). As a result, all ncRNA families analysed in this study represent evolutionarily conserved families with broad phylogenetic support. These curated CMs were used for all subsequent analyses.

### ncRNA copy number predictions across 60 genomes

Genome-wide ncRNA family prediction was performed using INFERNAL cmscan (v1.1.5) (Nawrocki and Eddy 2013), which identifies ncRNA genes by comparing genomic sequences to covariance models (CMs) that capture both sequence conservation and secondary structure. We used a curated set of 1,435 high-confidence CMs derived from Rfam, representing diverse and evolutionarily conserved ncRNA families (Ontiveros-Palacios et al. 2025). To ensure sensitivity and consistent detection across mammalian genomes, we applied the *--nohmm* option, which disables the initial Hidden Markov Model (HMM)-based prefilter and instead evaluates all candidate regions using full CM alignments. We also used the *--cut_ga* option to retain only predictions that meet family-specific gathering (GA) thresholds defined in Rfam, thereby ensuring biologically meaningful and high-confidence annotations. Additional filtering steps were applied to further improve reliability. For each species, we excluded predictions with E-values greater than 0.00001 or with alignment coverage below 80% of the CM model length, removing weak or partial matches. We also excluded ncRNA families detected in only a single species to reduce the influence of spurious lineage-specific predictions. These steps resulted in a final copy number matrix of 588 putative ncRNA families across 60 species (Table S2), comprising miRNA (*n* = 189), snRNA/snoRNA (*n* = 185), lncRNA (*n* = 152), cis-reg (*n* = 33), rRNA (*n* = 8), tRNA (*n* = 2), and miscellaneous RNA (*n* = 19). Because some loci are annotated by Rfam as both snRNA and snoRNA, reflecting overlapping roles in RNA processing, we treated them as a single combined category (Ontiveros-Palacios et al. 2025).

Overlaps among ncRNA predictions were assessed based on genomic coordinates using Bedtools (v2.31.1) (Quinlan And Hall 2010). A small proportion of annotations showed overlaps across species (mean 1.6% per species), primarily among large and small subunit rRNA (LSU and SSU), which are enriched in repetitive sequences, and in a few cases as pairs of miRNA annotations fully overlapping the same genomic locus on opposite strands. These were retained, as covariance model searches can assign structurally related RNAs to the same or overlapping loci, and such overlaps may represent distinct transcripts (e.g., embedded or antisense ncRNAs) (Carninci et al. 2005). All annotations meeting the filtering criteria were therefore kept. The complete set of ncRNA predictions for all 60 species (BED format) is provided in Table S3.

### Phylogenetic analyses of ncRNA copy numbers across 60 mammals

First, we tested for phylogenetic correlations between predicted ncRNA metrics (number of families, total copy number, and cumulative length) and genome characteristics (assembly size, total length, and BUSCO completeness score) using the *phytools* (v2.4.4) R package (Revell 2024). Trait covariances were estimated with the *phyl.vcv* function, which accounts for phylogenetic relatedness among species. A time-calibrated phylogeny of 60 mammals was obtained from TimeTree (v5) (Kumar et al. 2022). *P*-values were adjusted for multiple testing using the Benjamini-Hochberg method, and results with adjusted *P*-values < 0.05 were considered statistically significant.

Next, we analysed expansions and contractions of putative ncRNA family copy numbers across the time-calibrated phylogenetic tree described above using CAFE (v5) (Mendes et al. 2021). Prior to analysis, ncRNA families were filtered in two steps. First, families with extremely high copy numbers (> 100 copies in any species) were excluded to avoid inflating estimates of gene expansion and contraction rates. Second, from the remaining set, only families inferred to be present at the root of the mammalian phylogeny were retained, as required by the CAFE model. In practice, this corresponds to families detected in the early-diverging lineage (Monotremata) as well as in other clades. This resulted in a filtered copy number matrix of 291 putative ncRNA families across 60 species, which was used as input for the CAFE analysis.

Error models were applied to account for potential bias in copy number estimates due to genome assembly or annotation errors, and the family expansion and contraction rates were estimated separately for Monotremata, Marsupialia, and Eutheria based on the phylogenetic tree and observed ncRNA copy number. To ensure robust rate estimates, we performed 100 independent CAFE runs and retained the one with the highest log-likelihood as the best fit. Putative ncRNA families with adjusted *P*-values < 0.05 were considered to exhibit a significantly accelerated expansion or contraction rate along a given branch. In particular, we examined ncRNA family expansions and contractions across major mammalian clades, including Monotremata, Marsupialia, Atlantogenata, Euarchontoglires, and Laurasiatheria. ncRNA families showing significant expansions or contractions above the family level were visualised using the *circlize* (v0.4.16) R package (Gu et al. 2014).

To explore overall patterns of ncRNA family copy numbers across species, we also performed principal component analysis (PCA) on the same copy number dataset using the *prcomp* R package (Team 2010). Prior to PCA, copy numbers were log-transformed (log_2_[*n* + 1]) and z-score normalised to reduce the influence of high-copy, high-variance families. In addition, phylogenetic signal (Pagel’s λ) and evolutionary rate (σ^2^) of copy numbers were separately estimated for each of the putative 588 ncRNA families using the *phytools* R package (Revell 2024), based on the time-calibrated phylogenetic tree as mentioned above. *P*-values test the significance of phylogenetic signal and evolutionary rate, with adjusted *P*-values < 0.05 considered statistically significant. The relationship between phylogenetic signals and evolutionary rates across 588 ncRNA families was further visualised (Table S4). In addition, we examined the ncRNA families with consistent copy numbers across all mammals.

### Analyses of ncRNA families with variable and high copy numbers across species

Based on the evolutionary rates (σ^2^), we focused on ten ncRNA families with high variability and high copy numbers across species. To assess if duplicated copies may have lost functionality, we examined their overlap with transposable elements (TEs). Each of the 60 genomes was soft-masked using RepeatMasker (v4.1.2) with the default parameter settings (Smit 2013–2015), and for each ncRNA family, the proportion of copies overlapping with TEs was calculated. To explore potential mechanisms underlying high copy numbers, we examined the genomic distribution of these ten families across species and visualised the 5S rRNA gene loci in the *Lemur catta* genome using the *chromoMap* (v4.4.1) R package (Anand And Rodriguez Lopez 2022).

### Statistical analyses

The statistical analyses in this study, including phylogenetic correlation analysis, principal component analysis, phylogenetic signal estimation (Pagel’s λ), evolutionary rate calculation (σ^2^), hypergeometric test, and Mann-Whitney *U* tests were conducted in R (v4.5.1) (Team 2010). *P*-values were adjusted for multiple testing where applicable, and adjusted *P*-values < 0.05 were considered statistically significant unless specifically noted.

## Results

### Overview of predicted ncRNA families across 60 mammals

**Fig. 1:**
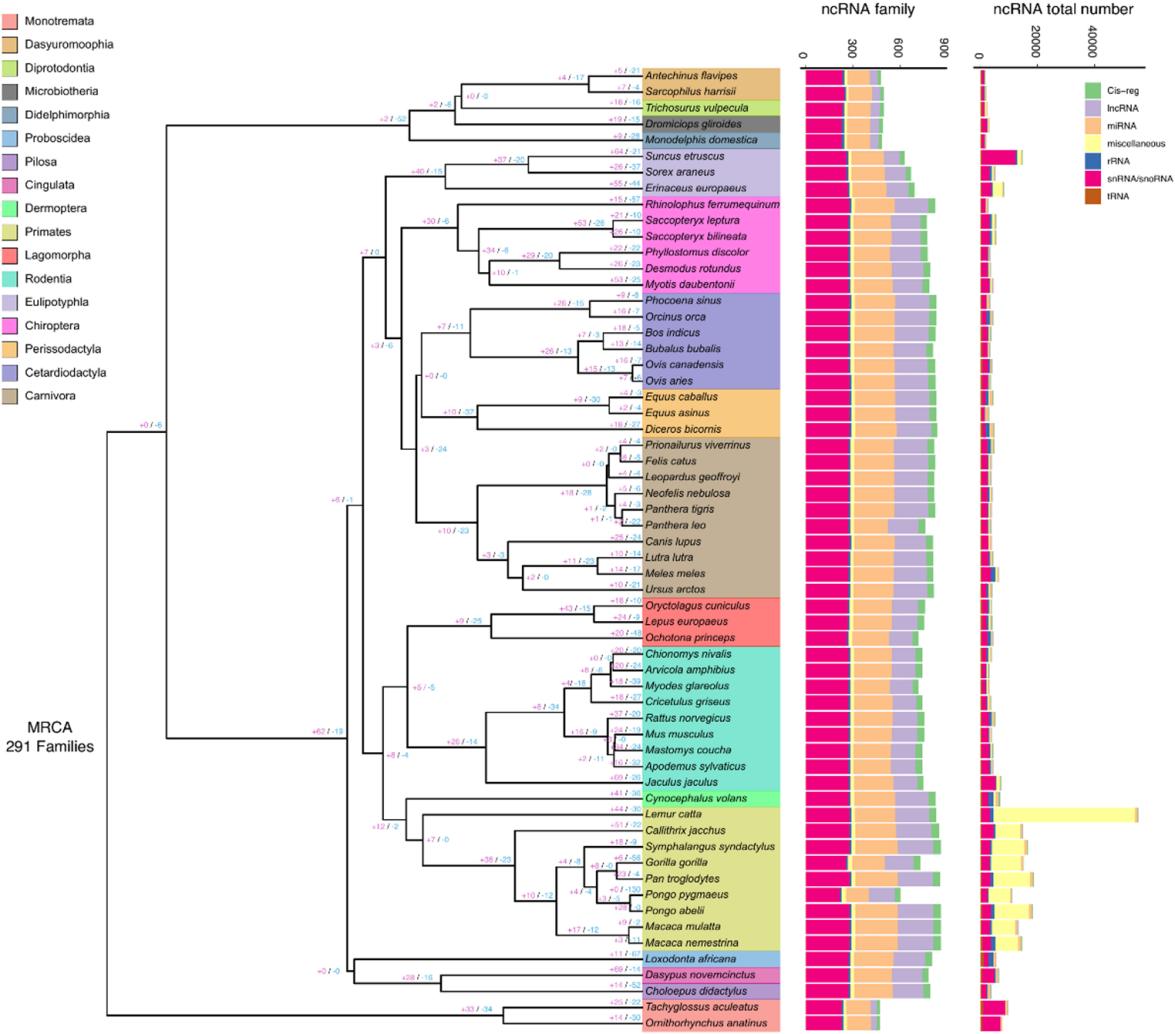
Distribution of ncRNA families across 60 mammalian genomes. The phylogeny was obtained from TimeTree v5. Numbers on the tree indicate the number of ncRNA families that have undergone expansion (purple) or contraction (blue) inferred by CAFE v5. The bar plots on the right show the total number of ncRNA families as well as the total ncRNA gene copies, identified using the Infernal pipeline based on 588 well-curated, conserved ncRNA families

Using the INFERNAL pipeline, we predicted copy numbers of 588 well-curated ncRNA families across 60 mammalian genomes. The number of putative ncRNA families per species ranges from 314 (*Ornithorhynchus anatinus* and *Tachyglossus aculeatus*) to 573 (*Macaca mulatta* and *Macaca nemestrina*), with an average of 432 ± 74 families (Fig. 1). Overall, eutherian mammals have significantly more ncRNA families than non-eutherians (*P* = 2.032 × 10^− 5^, Mann-Whitney *U* test). Within eutherians, primates exhibit significantly higher numbers of putative ncRNA families compared with other eutherian orders (*P* = 9.46 × 10^− 3^, Mann-Whitney *U* test). In terms of total ncRNA gene copy numbers, mammals exhibit considerable variation, ranging from 1,748 in *Antechinus flavipes* to 55,232 in *Lemur catta*. This variation is largely driven by lineage-specific expansions of a few ncRNA families, including *U6* in *Suncus etruscus*, miscellaneous ncRNA families such as *Metazoa_SRP* in primates, and *SNORA7* in Monotremata (Fig. 1).

**Fig. 2:**
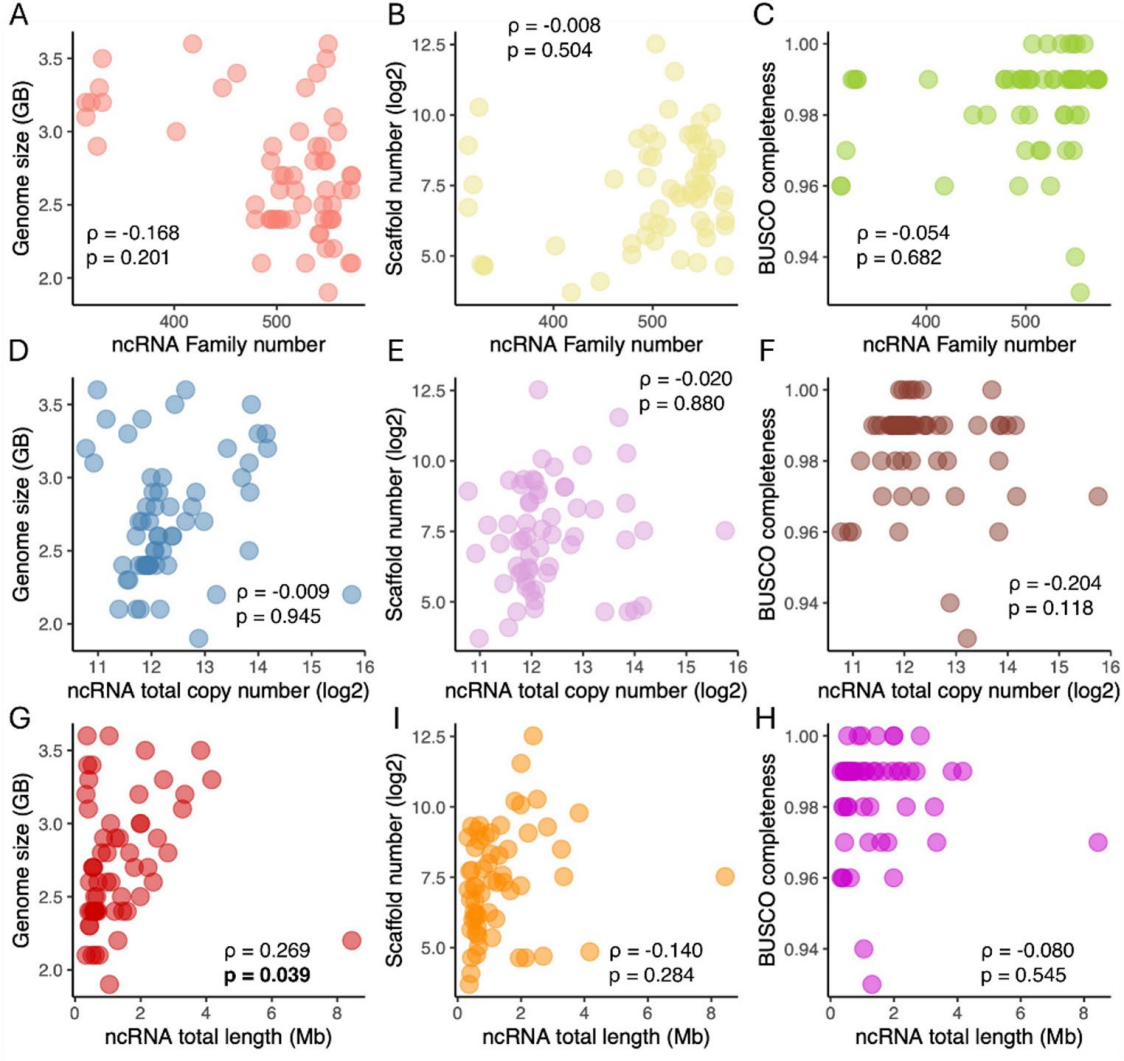
Phylogenetic correlations between ncRNA family and genome characteristics. **A–C** Correlations between the number of ncRNA families and genome size (GB), scaffold numbers and assembly completeness (BUSCO scores). **D–F** Correlations between total ncRNA gene copies and the same genome characteristics. **G–I** Correlations between cumulative ncRNA gene length (Mb) and the same genome characteristics. Phylogenetic correlation coefficients (ρ) and *P*-value were calculated using the R package *phytools*

Next, we assessed whether predicted ncRNA family number, total copy number, and cumulative length are associated with genomic characteristics using phylogenetic correlation analysis based on the established time-calibrated mammalian phylogeny (see Materials and Methods). Neither ncRNA family number nor total copy number showed significant correlations with genome size, scaffold number, or assembly completeness (Fig. 2; all *P-adj* > 0.05). Specifically, ncRNA family number was not significantly correlated with genome size (ρ = −0.168, *P-adj* = 0.201), scaffold number (ρ = −0.088, *P-adj* = 0.504), or assembly completeness (ρ = −0.054, *P-adj* = 0.682) (Fig. 2A–C). Similarly, ncRNA total copy number showed no statistically significant association with genome size (ρ = −0.009, *P-adj* = 0.945), scaffold number (ρ = −0.020, *P-adj* = 0.880), or assembly completeness (ρ = −0.204, *P-adj* = 0.118) (Fig. 2D–F). In contrast, ncRNA cumulative length exhibited significant positive correlation with genome size (ρ = 0.269, *P-adj* = 0.039), but not with scaffold number (ρ = −0.140, *P-adj* = 0.284) or BUSCO completeness (ρ = −0.080, *P-adj* = 0.545) (Fig. 2G–I). These results indicate that while ncRNA repertoire size (family number and total copy number) is largely independent of genome assembly characteristics, total ncRNA sequence content exhibits a modest positive association with genome size.

### Expansions and contractions of putative ncRNA family copy numbers across the mammalian phylogeny

**Fig. 3:**
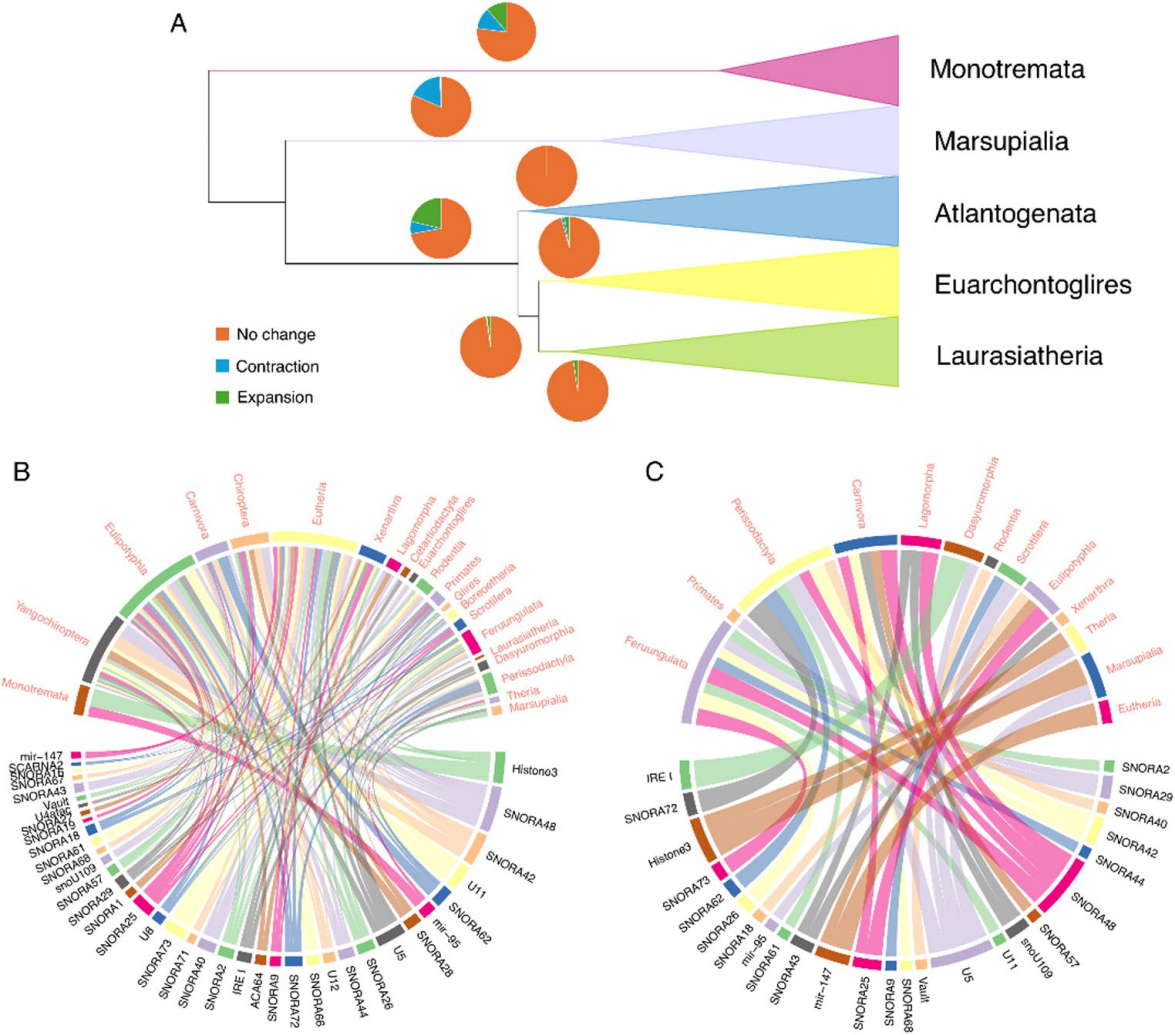
Expansion and contraction of ncRNA families across the mammalian phylogeny. **A** Evolutionary status of ncRNA families (expansion, contraction, or no change) in major clades. A total of 291 ncRNA families were inferred to be ancestral to mammals. Pie charts along the branches indicate family status. **B** ncRNA families showing significant expansions at taxonomic levels above the family level (*P* < 0.05). **C** ncRNA families showing significant contractions at taxonomic levels above the family level (*P* < 0.05). For **B** and **C**, lineages represented by a single species were excluded. Red text denotes clades, while black text indicates ncRNA families. Connections between them represent significant expansion or contraction of a given ncRNA family inferred at the ancestral node of the corresponding clade

Using CAFE analysis, we assessed the expansion and contraction of predicted ncRNA family copy numbers across the mammalian phylogeny. For this analysis, we retained 291 (49.5%) of 588 families that were predicted to be present at the ancestral node of mammals (see Materials and Methods). We observed that the birth-death rates of ncRNA families were higher in Eutheria (0.0039) compared with Monotremata (0.0022) and Marsupialia (0.0010), indicating more rapid family turnover in eutherian clades. This pattern is reflected in the number of families expanded or contracted at the ancestral nodes of these three clades: Eutheria (62 expansions; 19 contractions), Monotremata (33 expansions; 34 contractions), and Marsupialia (2 expansions; 52 contractions) (Fig. 1). By examining major clades, we noticed that the ancestral node of Eutheria experienced a greater number of family expansions compared with Monotremata and Marsupialia, whereas Marsupialia underwent a higher number of family contractions (Fig. 3A; Table S5). Most families were predicted to remain unchanged at the ancestral nodes of Atlantogenata, Boreoeutheria, Euarchontoglires, and Laurasiatheria, possibly due to their relatively short divergence times (Fig. 3A). Among the 291 putative ncRNA families examined, 42 showed significant copy-number expansions or contractions (*P* < 0.05) along at least one branch of the mammalian phylogeny. Above the family level, significant expansions were more common than significant contractions (Fig. 3B–C). Notably, several snoRNA (e.g., *SNORA2*, *SNORA40*) and snRNA (e.g., *U5*,* U8*) families were particularly dynamic, exhibiting multiple significant lineage-specific contractions or expansions across clades (Fig. 3B–C). Strikingly, all snoRNA families with significant copy-number shifts (*P* < 0.05) belonged to the H/ACA class (SNORA), whereas none of the C/D box class (SNORD) exhibited significant changes (Fig. 3B–C).

### Analysis of putative ncRNA family copy numbers

**Fig. 4:**
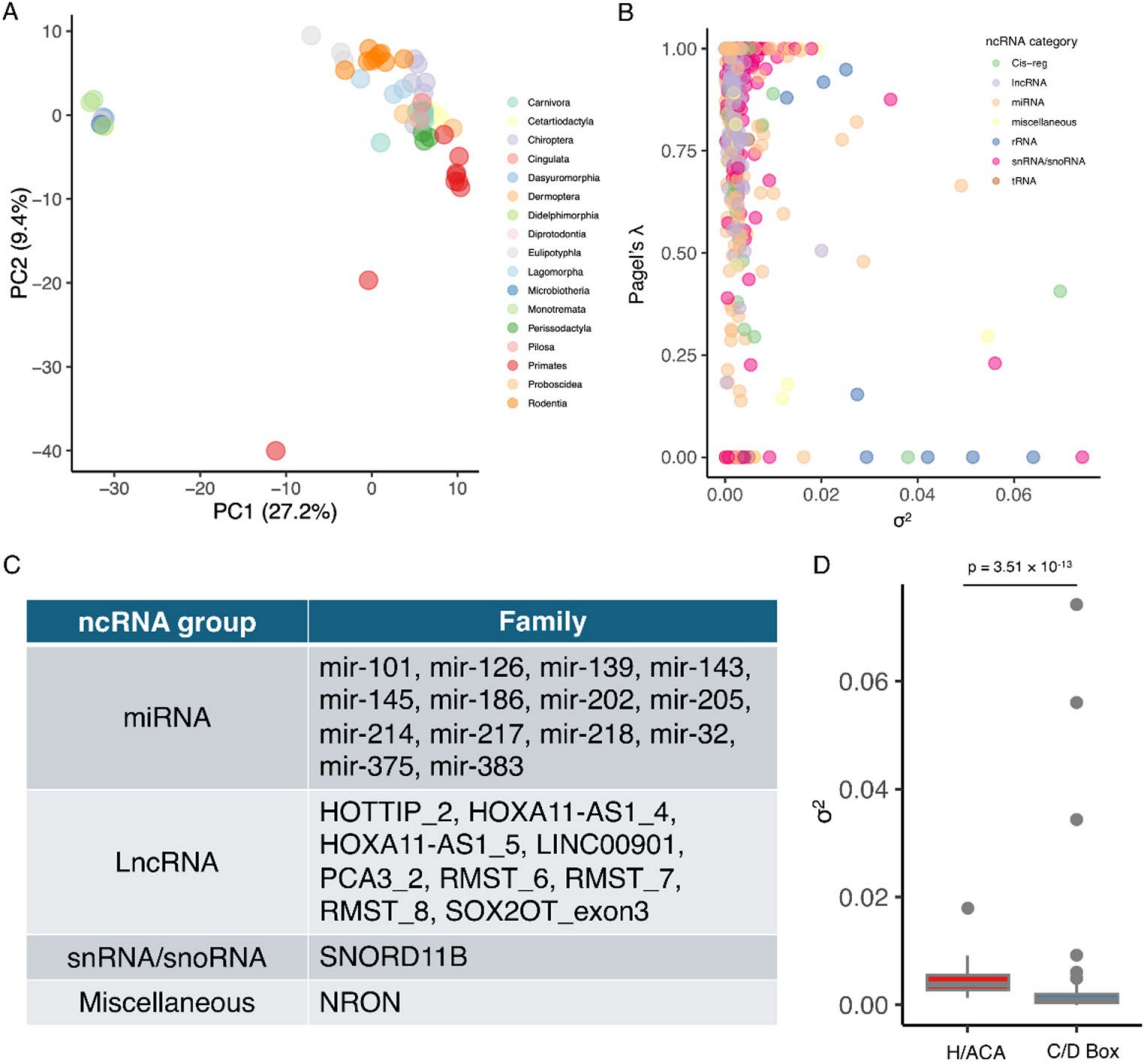
Analyses of ncRNA copy numbers across mammals. **A** Principal component analysis (PCA) of ncRNA numbers across 60 mammalian species. Copy numbers of 588 well-curated families across 60 mammals were log-transformed prior to the analysis. Colors indicate different mammalian orders. **B** Relationship between phylogenetic signals (Pagel’s λ) and evolutionary rate (σ²) across 588 ncRNA families, with colors indicating ncRNA categories. **C** A list of ncRNA families exhibiting invariable copy numbers across all 60 mammals examined. **D** Distributions of evolutionary rate estimates (σ²) for H/ACA (SNORA) and C/D box (SNORD) snoRNA families

To explore overall patterns of ncRNA family diversity across species, we performed a principal component analysis (PCA) based on total copy numbers of putative 588 families across all 60 species. The first two components clearly separated species into two clusters: non-eutherian mammals (Monotremata and Marsupialia) and eutherian mammals, with the exception of two primate species (*Pongo pygmaeus* and *Gorilla gorilla*) (Fig. 4A).

To further address the evolution of putative ncRNA family copy numbers, we evaluated the phylogenetic signal (Pagel’s λ) and evolutionary rate (σ^2^) for each family based on the established mammalian phylogeny (see Materials and Methods). Pagel’s λ quantifies the strength of phylogenetic signal in copy number distribution, ranging from 0 (no phylogenetic signal) and 1 (variation fully explained by phylogeny) (Pagel 1999), while σ^2^ reflects the rate of copy number evolution under a Brownian motion model, with higher values indicating faster rates of change (Garland et al. 1993). A large proportion of ncRNA families exhibited evolutionarily conserved copy numbers that mirror the mammalian phylogeny, as indicated by high Pagel’s λ and low σ^2^ values (Fig. 4B). We identified 25 out of 588 ncRNA families (4.25%) that exhibit invariable copy numbers across all 60 mammals examined, including 14 miRNA, 9 LncRNA, 1 snRNA/snoRNA and 1 miscellaneous ncRNA family (Fig. 4C). Among these, four miRNA families (mir-101, mir-126, mir-214, and mir-218) consistently occur in two copies across all species, whereas the remaining families are uniformly present as single-copy loci (Table S2).

In contrast, miRNA families were significantly enriched among those with low phylogenetic signal. Specifically, 45 out of the 92 ncRNA families with low Pagel’s λ values (0.1 ~ 0.7) were miRNA families, a significant over-representation (*P* = 1.97 × 10^− 4^, hypergeometric test) compared to the background frequency of 189 miRNA families among 588 total ncRNA families. Consistent with the CAFE results (Fig. 3B–C), H/ACA snoRNA families (SNORA) exhibited significantly higher evolutionary rate estimates (σ^2^) compared with C/D box snoRNAs (SNORD) (*P* = 3.51 × 10^− 13^, Mann-Whitney *U* test, Fig. 4D). In particular, several putative ncRNA families, such as *SNORD116*, *GP_knot1*, and *5_8S_rRNA* exhibited substantial variations in copy number across species (Table S2).

### Analysis of ncRNA families with variable and high copy numbers across species

**Fig. 5:**
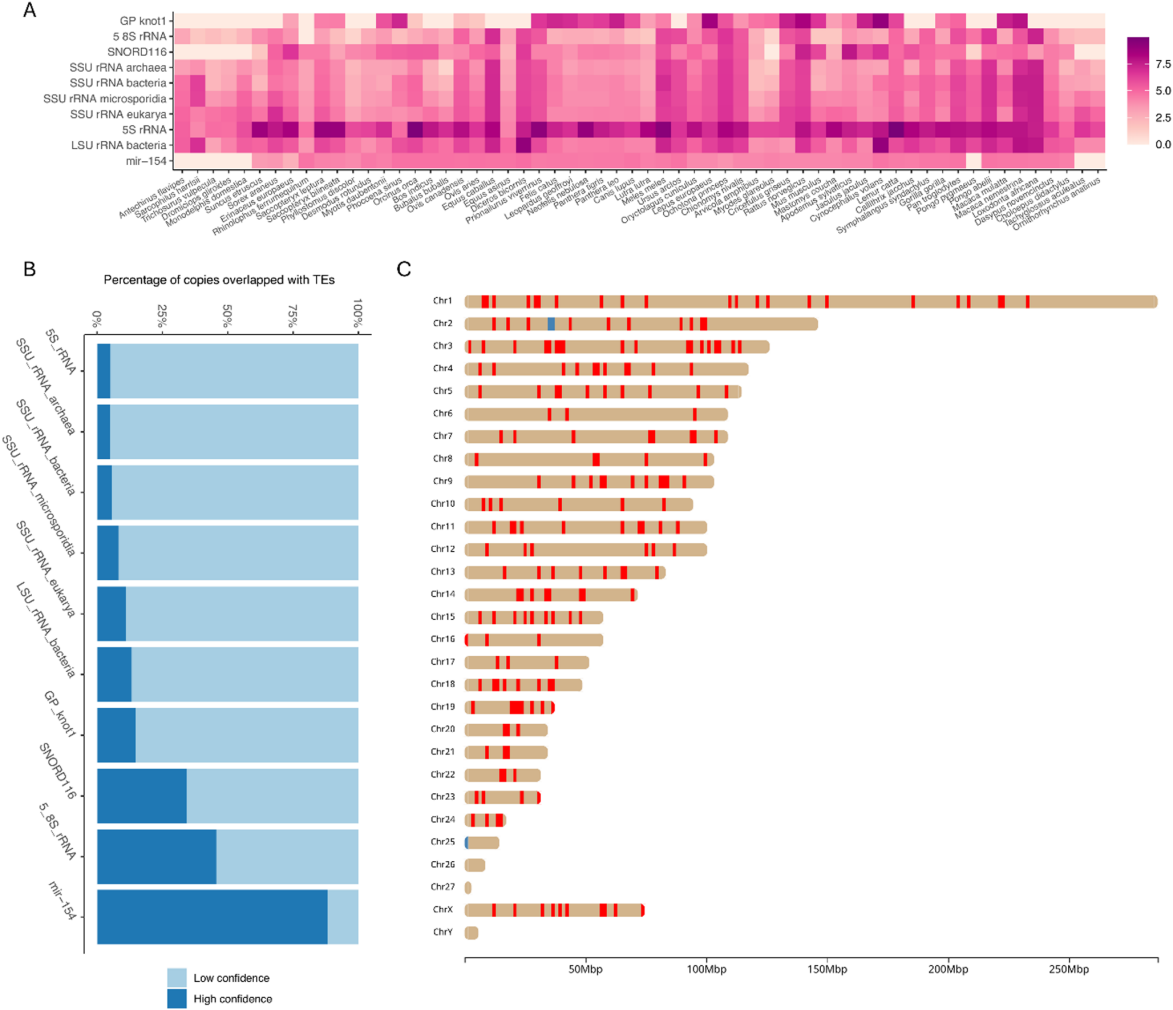
Analyses of ncRNA families exhibiting variable and high copy numbers across species. **A** Heatmap of copy numbers for 10 highly variable ncRNA families. Values are displayed on a log-transformed scale. **B** Proportion of high- versus low-confidence copies within the same 10 variable ncRNA families. Low-confidence copies are defined as ncRNAs overlapping annotated transposable elements (TEs). Copy numbers for each family were analysed across all species combined. **C** Genomic distribution of *5S_rRNA* copies in *Lemur catta*. Bars along the chromosomes represent scanning windows (~ 1.5 Mb) containing 5 S rRNA copies. Bar width is not proportional to copy number. Blue bars highlight regions with high copy numbers, likely resulting from tandem duplication

Next, we investigated the 10 putative ncRNA families exhibiting highly variable (based on evolutionary rate σ^2^) and high copy numbers across species. For example, the highest copy numbers of *5_8S_rRNA* were found in distantly-related lineages, including *Equus caballus*, *Loxodonta africana*, and *Pongo abelii* (Fig. 5A). *SNORD116* appeared to have undergone independent duplication events in multiple lineages, including species in Eulipotyphla, Perissodactyla, and Primates (Fig. 5A). We further observed that a large proportion of these duplicated copies overlap with annotated transposable elements (TEs), with the exception of *mir-154* (Fig. 5B).

To explore potential forces driving duplication, we investigated these 10 highly variable, high copy number ncRNA families and uncovered distinct patterns of duplication. For instance, across 60 species, *mir-154* and *SNORA116* copies are predominantly arranged as tandem duplications clustered in the same chromosome, while *5S_rRNA* copies are more widely dispersed across chromosomes, interspersed with regions of tandem duplications (Table S3). For example, the *Lemur catta* genome harbours the highest number of putative *5S_rRNA* copies among all species analysed. We found that most copies are scattered across the chromosomes, with the exception of chr26, chr27 and chrY (Fig. 5C). Noticeably, there are two large clusters of *5S_rRNA* duplicates on chr2 and chr25. The former comprises 100 copies within a 240 kb region, while the latter includes 144 copies within a 483 kb region (blue bars, Fig. 5C).

## Discussion

Despite their essential roles in gene regulation, ncRNA genes remain insufficiently annotated in most genomes, posting major challenges to reconstruct their evolutionary trajectories across the tree of life. In this study, using high-confidence CMs from Rfam we applied a unified approach (INFERNAL) to systematically predict and analyse 588 conserved ncRNA gene families across 60 mammalian genomes within a phylogenetic framework, providing one of the large-scale comparative perspectives on ncRNA copy number evolution. Nonetheless, even with stringent filtering criteria, the ncRNA families predicted in this study should be regarded as putative, as their functional relevance has not yet been experimentally verified. Importantly, our analyses focus on evolutionary dynamics at the ncRNA family level rather than on the functional status of individual copies, many of which may represent TE-derived or pseudogenised duplicates that nonetheless retain informative phylogenetic signal.

We observed large variation in ncRNA family numbers across mammals, with a noticeable increase on total copy number in primates (Fig. 1). This pattern is largely driven by the expansion of the miscellaneous ncRNA family Metazoa_SRP, which ranges from 6,798 copies in *Callithrix jacchus* to 48,336 in *Lemur catta* across primates, compared with only tens to a few hundred copies in non-primate species (Table S2), suggesting a genuine primate-specific expansion. In contrast, non-eutherian mammals appear to harbor fewer ncRNA families than eutherians (Fig. 1), although this pattern is likely influenced by methodological bias rather than true biological absence. Our analyses relied on curated covariance models (CMs) (Nawrocki and Eddy 2013), which are enriched for well-studied model organisms such as humans, leading to higher detection sensitivity for conserved ncRNA families and for species closely related to model organisms. Consequently, more divergent or lineage-specific ncRNA families, particularly in underrepresented lineages such as Monotremata and Marsupialia, may be underdetected. Consistent with this, our PCA revealed a clear separation between non-eutherian and eutherian groups (Fig. 4A), which is therefore likely driven, at least in part, by differential detection sensitivity rather than purely biological signals. Although our dataset is not exhaustive, the use of a uniform, structure-aware annotation framework across 60 species provides a robust basis for comparative analysis of conserved ncRNA families and their evolutionary dynamics.

Phylogenetic correlation analyses revealed no significant relationship between putative ncRNA family numbers or total copy numbers and genome size, scaffold number, or assembly completeness (Fig. 2), indicating that the observed variation is unlikely to be driven by genome characteristics alone. Notably, we identified 25 ncRNA families that exhibit invariable copy numbers across all 60 mammals (Fig. 4C), suggesting that these families may be subject to strong evolutionary constraint, potentially reflecting sensitivity to gene dosage or structural requirements that limit duplication and loss. However, copy number conservation alone does not indicate functional relevance, and further analyses would be required to assess their biological roles.

In contrast, we observed that the ancestral eutherian node exhibited the largest number of ncRNA family expansions across the phylogeny, particularly within snRNA (e.g. *U5*, *U8*, *U11*) and H/ACA snoRNAs (e.g. *SNORA42*, *SNORA71*) (Fig. 3A; Table S5). Similar expansion patterns have also been reported in humans and other mammals (Bergeron et al. 2021). These ncRNA classes are associated with core RNA processing pathways, including splicing and rRNA modification (Kufel And Grzechnik 2019; Peculis And Steitz 1993; Tarn And Steitz 1996), although the functional consequences of copy number variation remain unclear. Notably, most significant copy-number shifts occurred in H/ACA families, whereas C/D box snoRNA families showed little evidence of expansion or contraction across lineages above the family level (Fig. 3B–C). This asymmetry likely reflects differences in evolutionary constraint: C/D box snoRNAs typically target highly conserved rRNA methylation sites and are frequently embedded within conserved host genes (Fafard-Couture et al. 2025; Li et al. 2025), potentially limiting their tolerance to copy number changes. In contrast, H/ACA families appear to experience faster birth-death dynamics, consistent with previous observations of greater variability and turnover in their genomic distribution (Bergeron et al. 2021). Comparisons of evolutionary rate estimates between H/ACA and C/D box families provide additional support for this interpretation (Fig. 4D). Overall, these patterns suggest that ncRNA classes follow distinct evolutionary trajectories, with some families under strong constraint and others exhibiting greater flexibility in copy number evolution across mammalian lineages.

Contrary to the general pattern of ncRNA family expansions at ancestral nodes, we discovered that a subset of families underwent independent, lineage-specific duplications. Notably, 45 out of 92 ncRNA families with low phylogenetic signals (Pagel’s λ between 0.1 and 0.7) were miRNA families (Fig. 4B; *P* = 1.97 × 10^− 4^, hypergeometric test; Table S4), indicating a significant enrichment. Families with λ values close to 0 were excluded because some ncRNA families showed identical copy numbers across all species. In such cases, λ is estimated as zero not due to an absence of phylogenetic structure, but because no variation exists for the model to evaluate. This enrichment is consistent with the higher evolutionary plasticity of miRNAs, which could exhibit lineage-specific variation in copy number. Duplicated miRNAs can differ in sequence or expression context (Dexheimer And Cochella 2020), and lineage-specific expansions of miRNA families have been documented across many animal clades, including *mir-181* in vertebrates (Yang et al. 2014), *mir-421* in eutherians (Hertel et al. 2006), the primate-specific C19MC cluster (Noguer-Dance et al. 2010), and the rapidly evolving *mir-466* family in mouse (Boudreau et al. 2013). Consistent with previous findings (Yuan et al. 2011), we identified 15 putative copies of *mir-466* in mouse (Table S2), a family previously associated with TE-derived duplication such as SINE (short interspersed nuclear elements) and LTR (long terminal repeats) (Zheng et al. 2011). While such lineage-specific expansions may reflect diverse evolutionary processes, including duplication, sequence divergence, and turnover, their functional consequences remain uncertain. These miRNA families therefore represent useful candidates for future studies aimed at assessing the evolutionary and potential biological significance of lineage-specific ncRNA variation.

Examination of highly variable ncRNA families revealed contrasting duplication patterns, including tandem expansions (e.g., *mir-154* and *SNORA116*) and dispersed duplications (e.g., *5S_rRNA*). These observed patterns are consistent with distinct underlying mutational mechanisms and form the basis of our interpretation of ncRNA copy number evolution. Tandem duplications are likely driven by unequal recombination or replication slippage, which generate local copy number expansions within genomic clusters, whereas dispersed duplications may arise through segmental duplication or retrotransposition, often mediated by transposable elements such as SINEs and long interspersed nuclear elements (LINEs) (Bailey et al. 2002; Ohshima 2013). The substantial overlap between high-copy ncRNA families and transposable elements observed in our dataset (Fig. 5B) further supports a role for TE-mediated processes in shaping dispersed ncRNA repertoires. While distinguishing the relative contributions of these processes for individual ncRNA families remains challenging without detailed locus-level analyses, the observed diversity of duplication patterns suggests that multiple mutational processes contribute to the evolution of ncRNA copy number across mammals.

In this study, we systematically annotated 588 well-curated, conserved ncRNA families across 60 mammals and explored their copy number evolution. Our analyses revealed that, similar to other prediction tools (Zhang et al. 2017), ncRNA families predicted by the INFERNAL pipeline show a bias toward well-studied model organisms and their closely related species, likely reflecting the greater availability of experimental data for these taxa. We found that the eutherian ancestor underwent a burst of ncRNA expansions, largely involving snRNA and H/ACA snoRNA families, whereas miRNA families tend to experience lineage-specific turnover that may have driven distinct regulatory innovations. Finally, we demonstrated that high-copy ncRNA families may arise through distinct, and sometimes combined, duplication strategies. However, due to the intrinsic properties of ncRNAs, current computational pipelines remain limited in their ability to distinguish functional from nonfunctional copies, particularly among TE-derived families, and are unable to capture the context-dependent expression patterns that shape ncRNA function. Future work integrating state-of-the-art comparative genomics annotation pipelines with RNA sequencing, tissue- and stage-specific expression profiling, and targeted functional assays will be crucial for disentangling the evolutionary and regulatory significance of ncRNA families. Taken together, our findings highlight the diverse evolutionary trajectories of ncRNA family copy number across mammals and their potential to shape lineage-specific regulatory networks and organismal complexity.

## Supplementary Information

Below is the link to the electronic supplementary material.

Supplementary Material 1 (XLSX 13 KB)

Supplementary Material 1 (XLSX 137 KB)

Supplementary Material 1 (XLSX 14,393 KB)

Supplementary Material 1 (XLSX 33 KB)

Supplementary Material 1 (XLSX 19 KB)

## Data Availability

The accession numbers of the 60 genomes analysed in this study are provided in Supplementary Table S1. Predicted ncRNA family annotations in BED format and their putative copy numbers across all species are available in Supplementary Table S2 and S3, respectively. The code used for data analysis is available at: https://github.com/HuangLabUCD/ncRNA_CNV_project.

## References

[CR1] Abbott AL, Alvarez-Saavedra E, Miska EA, Lau NC, Bartel DP, Horvitz HR, Ambros V (2005) The let-7 MicroRNA family members mir-48, mir-84, and mir-241 function together to regulate developmental timing in *Caenorhabditis elegans*. Dev Cell 9:403–414 16139228 10.1016/j.devcel.2005.07.009 PMC3969732

[CR2] Anand L, Rodriguez Lopez CM (2022) ChromoMap: an R package for interactive visualization of multi-omics data and annotation of chromosomes. BMC Bioinform 23:33 10.1186/s12859-021-04556-z PMC8753883 35016614

[CR3] Bailey JA, Gu Z, Clark RA, Reinert K, Samonte RV, Schwartz S, Adams MD, Myers EW, Li PW, Eichler EE (2002) Recent segmental duplications in the human genome. Science 297:1003–1007 12169732 10.1126/science.1072047

[CR4] Bergeron D, Laforest C, Carpentier S, Calve A, Fafard-Couture E, Deschamps-Francoeur G, Scott MS (2021) SnoRNA copy regulation affects family size, genomic location and family abundance levels. BMC Genomics 22:414 34090325 10.1186/s12864-021-07757-1 PMC8178906

[CR5] Bieth E, Eddiry S, Gaston V, Lorenzini F, Buffet A, Conte Auriol F, Molinas C, Cailley D, Rooryck C, Arveiler B, Cavaille J, Salles JP, Tauber M (2015) Highly restricted deletion of the SNORD116 region is implicated in Prader-Willi Syndrome. Eur J Hum Genet 23:252–255 24916642 10.1038/ejhg.2014.103 PMC4297892

[CR6] Boudreau RL, Spengler RM, Hylock RH, Kusenda BJ, Davis HA, Eichmann DA, Davidson BL (2013) siSPOTR: a tool for designing highly specific and potent siRNAs for human and mouse. Nucleic Acids Res 41:e9 22941647 10.1093/nar/gks797 PMC3592398

[CR7] Carninci P, Kasukawa T, Katayama S, Gough J, Frith MC, Maeda N, Oyama R, Ravasi T, Lenhard B, Wells C, Kodzius R, Shimokawa K, Bajic VB, Brenner SE, Batalov S, Forrest AR, Zavolan M, Davis MJ, Wilming LG, Aidinis V, Allen JE, Ambesi-Impiombato A, Apweiler R, Aturaliya RN, Bailey TL, Bansal M, Baxter L, Beisel KW, Bersano T, Bono H, Chalk AM, Chiu KP, Choudhary V, Christoffels A, Clutterbuck DR, Crowe ML, Dalla E, Dalrymple BP, de Bono B, Della Gatta G, di Bernardo D, Down T, Engstrom P, Fagiolini M, Faulkner G, Fletcher CF, Fukushima T, Furuno M, Futaki S, Gariboldi M, Georgii-Hemming P, Gingeras TR, Gojobori T, Green RE, Gustincich S, Harbers M, Hayashi Y, Hensch TK, Hirokawa N, Hill D, Huminiecki L, Iacono M, Ikeo K, Iwama A, Ishikawa T, Jakt M, Kanapin A, Katoh M, Kawasawa Y, Kelso J, Kitamura H, Kitano H, Kollias G, Krishnan SP, Kruger A, Kummerfeld SK, Kurochkin IV, Lareau LF, Lazarevic D, Lipovich L, Liu J, Liuni S, McWilliam S, Madan Babu M, Madera M, Marchionni L, Matsuda H, Matsuzawa S, Miki H, Mignone F, Miyake S, Morris K, Mottagui-Tabar S, Mulder N, Nakano N, Nakauchi H, Ng P, Nilsson R, Nishiguchi S, Nishikawa S, Nori F, Ohara O, Okazaki Y, Orlando V, Pang KC, Pavan WJ, Pavesi G, Pesole G, Petrovsky N, Piazza S, Reed J, Reid JF, Ring BZ, Ringwald M, Rost B, Ruan Y, Salzberg SL, Sandelin A, Schneider C, Schonbach C, Sekiguchi K, Semple CA, Seno S, Sessa L, Sheng Y, Shibata Y, Shimada H, Shimada K, Silva D, Sinclair B, Sperling S, Stupka E, Sugiura K, Sultana R, Takenaka Y, Taki K, Tammoja K, Tan SL, Tang S, Taylor MS, Tegner J, Teichmann SA, Ueda HR, van Nimwegen E, Verardo R, Wei CL, Yagi K, Yamanishi H, Zabarovsky E, Zhu S, Zimmer A, Hide W, Bult C, Grimmond SM, Teasdale RD, Liu ET, Brusic V, Quackenbush J, Wahlestedt C, Mattick JS, Hume DA, Kai C, Sasaki D, Tomaru Y, Fukuda S, Kanamori-Katayama M, Suzuki M, Aoki J, Arakawa T, Iida J, Imamura K, Itoh M, Kato T, Kawaji H, Kawagashira N, Kawashima T, Kojima M, Kondo S, Konno H, Nakano K, Ninomiya N, Nishio T, Okada M, Plessy C, Shibata K, Shiraki T, Suzuki S, Tagami M, Waki K, Watahiki A, Okamura-Oho Y, Suzuki H, Kawai J, Hayashizaki Y, Consortium F (2005) Group RGER, genome science G the transcriptional landscape of the mammalian genome. Science 309, 1559–1563 10.1126/science.1112014 16141072

[CR8] Chan PP, Lin BY, Mak AJ, Lowe TM (2021) tRNAscan-SE 2.0: improved detection and functional classification of transfer RNA genes. Nucleic Acids Res 49:9077–9096 34417604 10.1093/nar/gkab688 PMC8450103

[CR9] Christmas MJ, Kaplow IM, Genereux DP, Dong MX, Hughes GM, Li X, Sullivan PF, Hindle AG, Andrews G, Armstrong JC, Bianchi M, Breit AM, Diekhans M, Fanter C, Foley NM, Goodman DB, Goodman L, Keough KC, Kirilenko B, Kowalczyk A, Lawless C, Lind AL, Meadows JRS, Moreira LR, Redlich RW, Ryan L, Swofford R, Valenzuela A, Wagner F, Wallerman O, Brown AR, Damas J, Fan K, Gatesy J, Grimshaw J, Johnson J, Kozyrev SV, Lawler AJ, Marinescu VD, Morrill KM, Osmanski A, Paulat NS, Phan BN, Reilly SK, Schaffer DE, Steiner C, Supple MA, Wilder AP, Wirthlin ME, Xue JR, Zoonomia Consortium, Birren BW, Gazal S, Hubley RM, Koepfli KP, Marques-Bonet T, Meyer WK, Nweeia M, Sabeti PC, Shapiro B, Smit AFA, Springer MS, Teeling EC, Weng Z, Hiller M, Levesque DL, Lewin HA, Murphy WJ, Navarro A, Paten B, Pollard KS, Ray DA, Ruf I, Ryder OA, Pfenning AR, Lindblad-Toh K, Karlsson EK (2023) Evolutionary constraint and innovation across hundreds of placental mammals. Science 380:eabn3943 37104599 10.1126/science.abn3943 PMC10250106

[CR10] Dexheimer PJ, Cochella L (2020) MicroRNAs: from mechanism to organism. Front Cell Dev Biol 8:409 32582699 10.3389/fcell.2020.00409 PMC7283388

[CR11] Doolittle WF (2013) Is junk DNA bunk? A critique of ENCODE. Proc Natl Acad Sci U S A 110:5294–5300 23479647 10.1073/pnas.1221376110 PMC3619371

[CR12] Dulai KS, von Dornum M, Mollon JD, Hunt DM (1999) The evolution of trichromatic color vision by opsin gene duplication in New World and Old World primates. Genome Res 9:629–638 10413401

[CR13] Eddy SR (2001) Non-coding RNA genes and the modern RNA world. Nat Rev Genet 2:919–929 11733745 10.1038/35103511

[CR14] Fafard-Couture E, Boulanger C, Faucher-Giguere L, Sinagoga V, Berthoumieux M, Hedjam J, Marcel V, Durand S, Bayfield MA, Bachand F, Abou Elela S, Jacques PE, Scott MS (2025) SnoBIRD: a tool to identify C/D box snoRNAs and refine their annotation across all eukaryotes. Nucleic Acids Res 53:gkaf708 40737086 10.1093/nar/gkaf708 PMC12309372

[CR15] Garland T Jr., Dickerman AW, Janis CM, Jones JA (1993) Phylogenetic Analysis of Covariance by Computer Simulation. Syst Biol 42:265–292

[CR16] Gu Z, Gu L, Eils R, Schlesner M, Brors B (2014) Circlize implements and enhances circular visualization in R. Bioinformatics 30:2811–2812 24930139 10.1093/bioinformatics/btu393

[CR17] Hertel J, Lindemeyer M, Missal K, Fried C, Tanzer A, Flamm C, Hofacker IL, Stadler PF, Students of Bioinformatics Computer Labs (2006) The expansion of the metazoan microRNA repertoire. BMC Genomics 7:25 16480513 10.1186/1471-2164-7-25 PMC1388199

[CR18] Hertel J, Bartschat S, Wintsche A, Otto C, Stadler PF (2012) Students of the Bioinformatics Computer L, Stadler PF (2012) Evolution of the let-7 microRNA family. RNA Biol 9:231–241 22617875 10.4161/rna.18974 PMC3384580

[CR19] Herzing LB, Romer JT, Horn JM, Ashworth A (1997) *Xist* has properties of the X-chromosome inactivation centre. Nature 386:272–275 9069284 10.1038/386272a0

[CR20] Hughes LA, Rudler DL, Siira SJ, McCubbin T, Raven SA, Browne JM, Ermer JA, Rientjes J, Rodger J, Marcellin E, Rackham O, Filipovska A (2023) Copy number variation in tRNA isodecoder genes impairs mammalian development and balanced translation. Nat Commun 14:2210 37072429 10.1038/s41467-023-37843-9 PMC10113395

[CR21] Innan H, Kondrashov F (2010) The evolution of gene duplications: classifying and distinguishing between models. Nat Rev Genet 11:97–108 20051986 10.1038/nrg2689

[CR22] Kang YJ, Yang DC, Kong L, Hou M, Meng YQ, Wei L, Gao G (2017) CPC2: a fast and accurate coding potential calculator based on sequence intrinsic features. Nucleic Acids Res 45:W12–W16 28521017 10.1093/nar/gkx428 PMC5793834

[CR23] Kufel J, Grzechnik P (2019) Small nucleolar RNAs tell a different tale. Trends Genet 35:104–117 30563726 10.1016/j.tig.2018.11.005

[CR24] Kumar S, Suleski M, Craig JM, Kasprowicz AE, Sanderford M, Li M, Stecher G, Hedges SB (2022) TimeTree 5: an expanded resource for species divergence times. Mol Biol Evol 39:msac174 35932227 10.1093/molbev/msac174 PMC9400175

[CR25] Lander ES, Linton LM, Birren B, Nusbaum C, Zody MC, Baldwin J, Devon K, Dewar K, Doyle M, FitzHugh W, Funke R, Gage D, Harris K, Heaford A, Howland J, Kann L, Lehoczky J, LeVine R, McEwan P, McKernan K, Meldrim J, Mesirov JP, Miranda C, Morris W, Naylor J, Raymond C, Rosetti M, Santos R, Sheridan A, Sougnez C, Stange-Thomann Y, Stojanovic N, Subramanian A, Wyman D, Rogers J, Sulston J, Ainscough R, Beck S, Bentley D, Burton J, Clee C, Carter N, Coulson A, Deadman R, Deloukas P, Dunham A, Dunham I, Durbin R, French L, Grafham D, Gregory S, Hubbard T, Humphray S, Hunt A, Jones M, Lloyd C, McMurray A, Matthews L, Mercer S, Milne S, Mullikin JC, Mungall A, Plumb R, Ross M, Shownkeen R, Sims S, Waterston RH, Wilson RK, Hillier LW, McPherson JD, Marra MA, Mardis ER, Fulton LA, Chinwalla AT, Pepin KH, Gish WR, Chissoe SL, Wendl MC, Delehaunty KD, Miner TL, Delehaunty A, Kramer JB, Cook LL, Fulton RS, Johnson DL, Minx PJ, Clifton SW, Hawkins T, Branscomb E, Predki P, Richardson P, Wenning S, Slezak T, Doggett N, Cheng JF, Olsen A, Lucas S, Elkin C, Uberbacher E, Frazier M, Gibbs RA, Muzny DM, Scherer SE, Bouck JB, Sodergren EJ, Worley KC, Rives CM, Gorrell JH, Metzker ML, Naylor SL, Kucherlapati RS, Nelson DL, Weinstock GM, Sakaki Y, Fujiyama A, Hattori M, Yada T, Toyoda A, Itoh T, Kawagoe C, Watanabe H, Totoki Y, Taylor T, Weissenbach J, Heilig R, Saurin W, Artiguenave F, Brottier P, Bruls T, Pelletier E, Robert C, Wincker P, Smith DR, Doucette-Stamm L, Rubenfield M, Weinstock K, Lee HM, Dubois J, Rosenthal A, Platzer M, Nyakatura G, Taudien S, Rump A, Yang H, Yu J, Wang J, Huang G, Gu J, Hood L, Rowen L, Madan A, Qin S, Davis RW, Federspiel NA, Abola AP, Proctor MJ, Myers RM, Schmutz J, Dickson M, Grimwood J, Cox DR, Olson MV, Kaul R, Raymond C, Shimizu N, Kawasaki K, Minoshima S, Evans GA, Athanasiou M, Schultz R, Roe BA, Chen F, Pan H, Ramser J, Lehrach H, Reinhardt R, McCombie WR, de la Bastide M, Dedhia N, Blocker H, Hornischer K, Nordsiek G, Agarwala R, Aravind L, Bailey JA, Bateman A, Batzoglou S, Birney E, Bork P, Brown DG, Burge CB, Cerutti L, Chen HC, Church D, Clamp M, Copley RR, Doerks T, Eddy SR, Eichler EE, Furey TS, Galagan J, Gilbert JG, Harmon C, Hayashizaki Y, Haussler D, Hermjakob H, Hokamp K, Jang W, Johnson LS, Jones TA, Kasif S, Kaspryzk A, Kennedy S, Kent WJ, Kitts P, Koonin EV, Korf I, Kulp D, Lancet D, Lowe TM, McLysaght A, Mikkelsen T, Moran JV, Mulder N, Pollara VJ, Ponting CP, Schuler G, Schultz J, Slater G, Smit AF, Stupka E, Szustakowki J, Thierry-Mieg D, Thierry-Mieg J, Wagner L, Wallis J, Wheeler R, Williams A, Wolf YI, Wolfe KH, Yang SP, Yeh RF, Collins F, Guyer MS, Peterson J, Felsenfeld A, Wetterstrand KA, Patrinos A, Morgan MJ, de Jong P, Catanese JJ, Osoegawa K, Shizuya H, Choi S, Chen YJ, Szustakowki J (2001) International human genome sequencing C (2001) Initial sequencing and analysis of the human genome. Nature 409:860–921 11237011 10.1038/35057062

[CR26] Li M, Liang C (2022) LncDC: a machine learning-based tool for long non-coding RNA detection from RNA-Seq data. Sci Rep 12:19083 36351980 10.1038/s41598-022-22082-7 PMC9646749

[CR27] Li Y, Chen X, Xiao S, Wang H, Li B, Zhang M, Wang K (2025) Unlocking the life code: a review of SnoRNA functional diversity and disease relevance. Cell Commun Signal 23:266 40468441 10.1186/s12964-025-02274-0 PMC12135493

[CR28] Mendes FK, Vanderpool D, Fulton B, Hahn MW (2021) CAFE 5 models variation in evolutionary rates among gene families. Bioinformatics 36:5516–5518 33325502 10.1093/bioinformatics/btaa1022

[CR29] Mogilyansky E, Rigoutsos I (2013) The miR-17/92 cluster: a comprehensive update on its genomics, genetics, functions and increasingly important and numerous roles in health and disease. Cell Death Differ 20:1603–1614 24212931 10.1038/cdd.2013.125 PMC3824591

[CR30] Nawrocki EP, Eddy SR (2013) Infernal 1.1: 100-fold faster RNA homology searches. Bioinformatics 29:2933–2935 24008419 10.1093/bioinformatics/btt509 PMC3810854

[CR31] Noguer-Dance M, Abu-Amero S, Al-Khtib M, Lefevre A, Coullin P, Moore GE, Cavaille J (2010) The primate-specific microRNA gene cluster (C19MC) is imprinted in the placenta. Hum Mol Genet 19:3566–3582 20610438 10.1093/hmg/ddq272

[CR32] Ohshima K (2013) RNA-mediated gene duplication and retroposons: retrogenes, LINEs, SINEs, and sequence specificity. Int J Evol Biol 2013:424726 23984183 10.1155/2013/424726 PMC3747384

[CR33] Ontiveros-Palacios N, Cooke E, Nawrocki EP, Triebel S, Marz M, Rivas E, Griffiths-Jones S, Petrov AI, Bateman A, Sweeney B (2025) Rfam 15: RNA families database in 2025. Nucleic Acids Res 53:D258–D267 39526405 10.1093/nar/gkae1023 PMC11701678

[CR34] Pagel M (1999) Inferring the historical patterns of biological evolution. Nature 401:877–884 10553904 10.1038/44766

[CR35] Peculis BA, Steitz JA (1993) Disruption of U8 nucleolar snRNA inhibits 5.8S and 28S rRNA processing in the Xenopus oocyte. Cell 73:1233–1245 8513505 10.1016/0092-8674(93)90651-6

[CR36] Piovesan A, Caracausi M, Antonaros F, Pelleri MC, Vitale L (2016) GeneBase 1.1: a tool to summarize data from NCBI gene datasets and its application to an update of human gene statistics. Database (Oxford) 2016 10.1093/database/baw153 PMC5199132 28025344

[CR37] Quinlan AR, Hall IM (2010) BEDTools: a flexible suite of utilities for comparing genomic features. Bioinformatics 26:841–842 20110278 10.1093/bioinformatics/btq033 PMC2832824

[CR38] Revell LJ (2024) phytools 2.0: an updated R ecosystem for phylogenetic comparative methods (and other things). PeerJ 12:e16505 38192598 10.7717/peerj.16505 PMC10773453

[CR39] Simao FA, Waterhouse RM, Ioannidis P, Kriventseva EV, Zdobnov EM (2015) BUSCO: assessing genome assembly and annotation completeness with single-copy orthologs. Bioinformatics 31:3210–3212 26059717 10.1093/bioinformatics/btv351

[CR40] Smit AH, Green R (2015) P (2013– RepeatMasker Open-4.0

[CR41] Storz JF (2016) Gene duplication and evolutionary innovations in hemoglobin-oxygen transport. Physiol (Bethesda) 31:223–232 10.1152/physiol.00060.2015 PMC5005275 27053736

[CR42] Tarn WY, Steitz JA (1996) A novel spliceosome containing U11, U12, and U5 snRNPs excises a minor class (AT-AC) intron in vitro. Cell 84:801–811 8625417 10.1016/s0092-8674(00)81057-0

[CR43] Team RDC (2010) R: A language and environment for statistical computing. R Foundation for Statistical Computing, Vienna, Austria

[CR44] Testa U, Pelosi E, Castelli G, Labbaye C (2017) miR-146 and miR-155: two key modulators of immune response and tumor development. Noncoding RNA. 10.3390/ncrna3030022 29657293 10.3390/ncrna3030022 PMC5831915

[CR45] Umu SU, Paynter VM, Trondsen H, Buschmann T, Rounge TB, Peterson KJ, Fromm B (2023) Accurate microRNA annotation of animal genomes using trained covariance models of curated microRNA complements in MirMachine. Cell Genom 3:100348 37601971 10.1016/j.xgen.2023.100348 PMC10435380

[CR46] Yang Z, Wan X, Gu Z, Zhang H, Yang X, He L, Miao R, Zhong Y, Zhao H (2014) Evolution of the mir-181 microRNA family. Comput Biol Med 52:82–87 25016292 10.1016/j.compbiomed.2014.06.004

[CR47] Yuan Z, Sun X, Liu H, Xie J (2011) MicroRNA genes derived from repetitive elements and expanded by segmental duplication events in mammalian genomes. PLoS One 6:e17666 21436881 10.1371/journal.pone.0017666 PMC3059204

[CR48] Zhang Y, Huang H, Zhang D, Qiu J, Yang J, Wang K, Zhu L, Fan J, Yang J (2017) A review on recent computational methods for predicting noncoding RNAs. Biomed Res Int 2017:9139504 28553651 10.1155/2017/9139504 PMC5434267

[CR49] Zheng GX, Ravi A, Gould GM, Burge CB, Sharp PA (2011) Genome-wide impact of a recently expanded microRNA cluster in mouse. Proc Natl Acad Sci U S A 108:15804–15809 21911408 10.1073/pnas.1112772108 PMC3179086

